# Supplementary material for: Hallmarks of Basidiomycete Soft- and White-Rot in Wood-Decay -Omics Data of Two Armillaria Species
Source: Microorganisms. 2021 Jan 11;9(1):149. doi: 10.3390/microorganisms9010149 (PMC7827401; doi:10.3390/microorganisms9010149)

LogFC for 113 common proteins in Trancriptomics and Proteomics for MvsNIM - Armillaria ostoyae

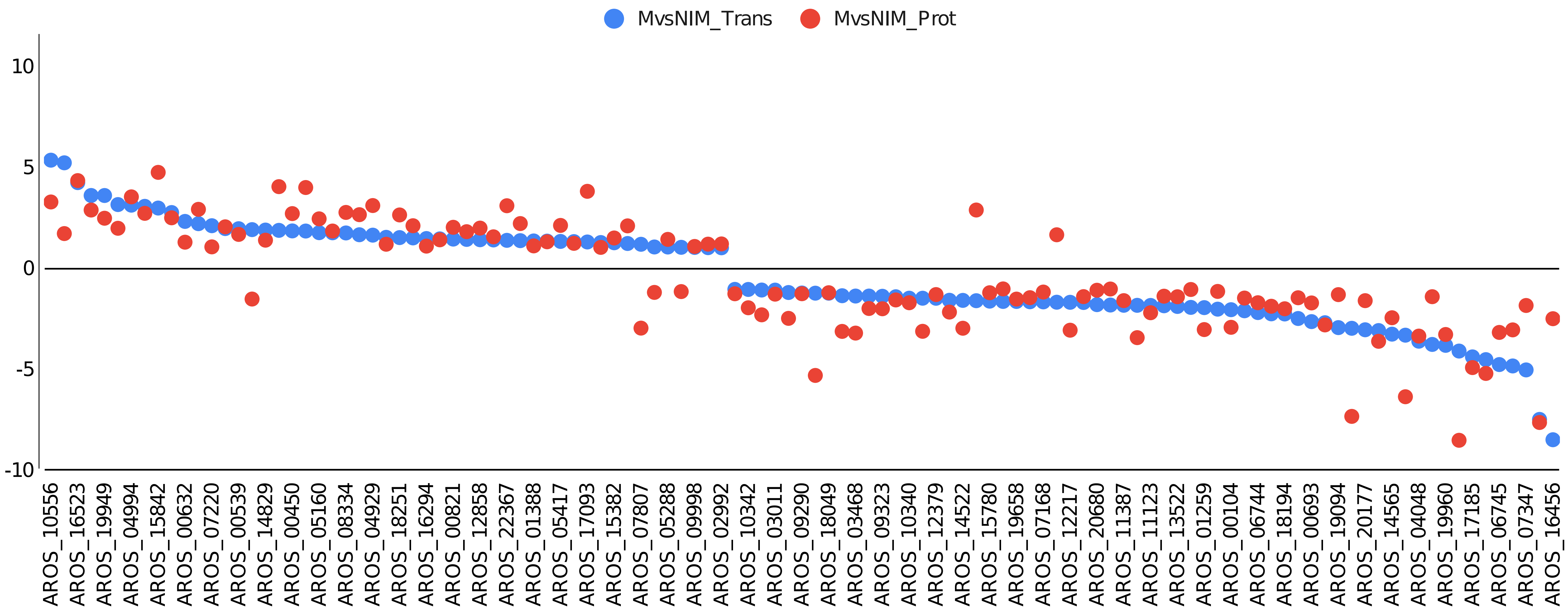

LogFC for 161 common proteins in Trancriptomics and Proteomics for MvsNIM - Armillaria cepistipes

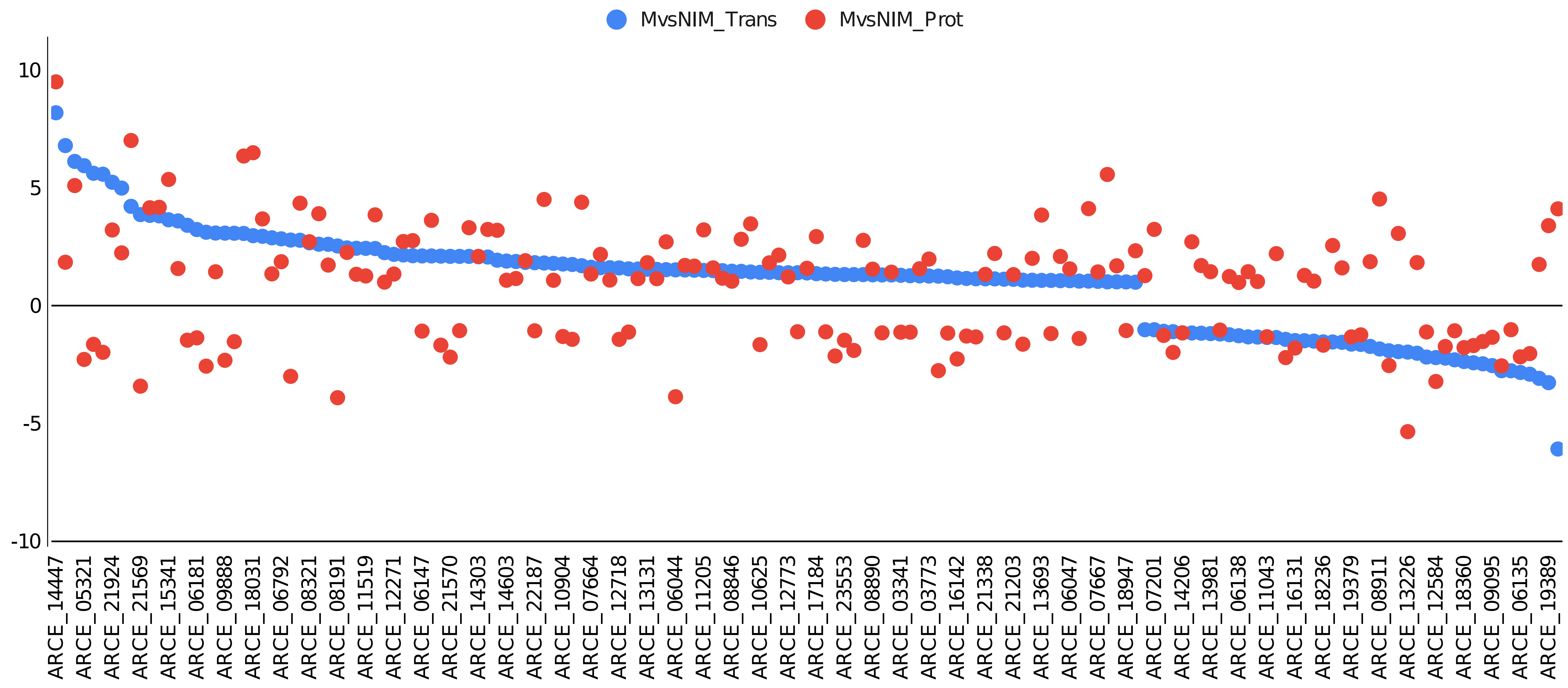

Supplement: Supplementary file 1 [file microorganisms-09-00149-s001.zip › Supplementary Figure 2.pdf]
