# Supplementary material for: Hallmarks of Basidiomycete Soft- and White-Rot in Wood-Decay -Omics Data of Two Armillaria Species
Source: Microorganisms. 2021 Jan 11;9(1):149. doi: 10.3390/microorganisms9010149 (PMC7827401; doi:10.3390/microorganisms9010149)
Supplement: Supplementary file 1 [file microorganisms-09-00149-s001.zip › Supplementary Figure 5.pdf]

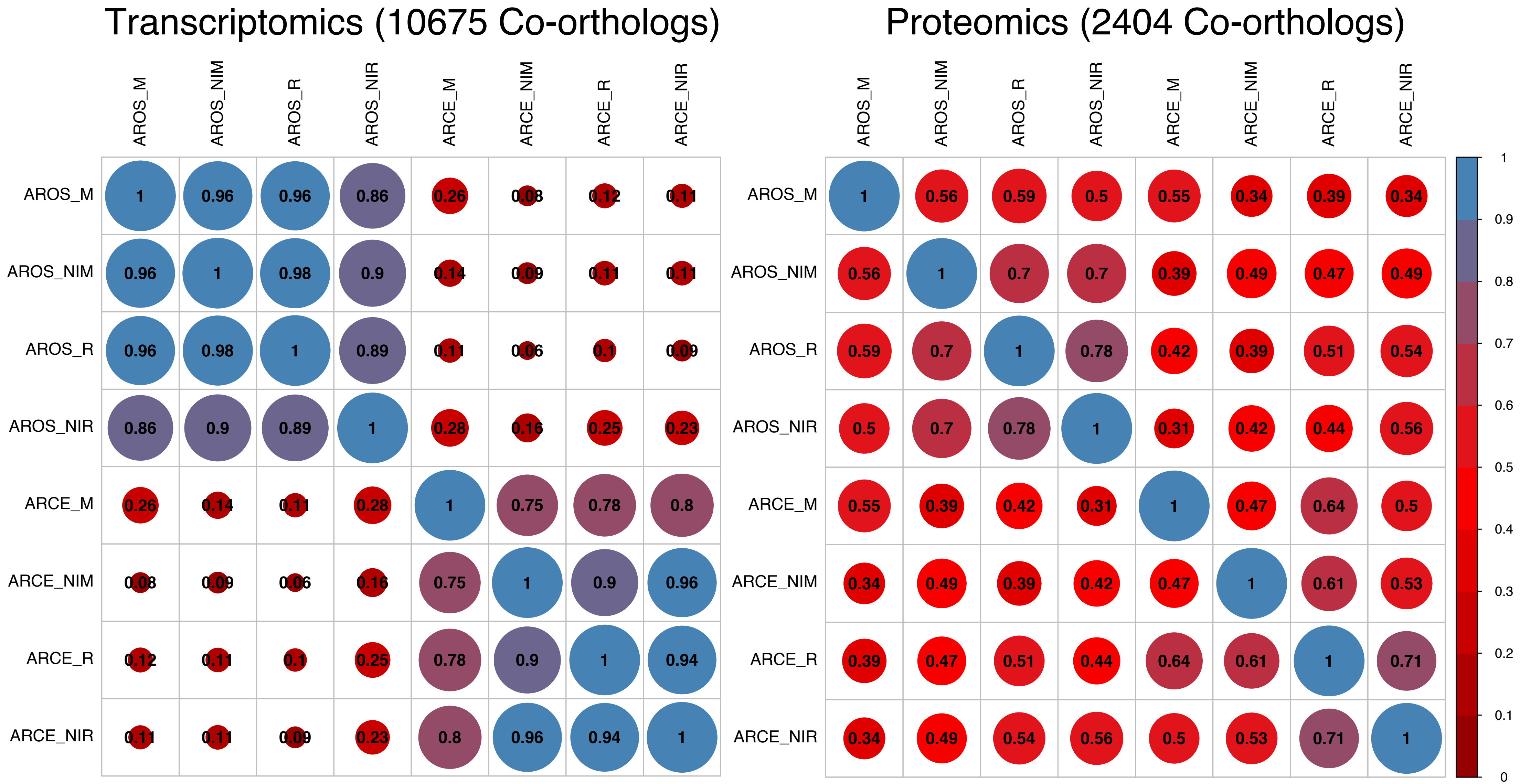


All co-orthologs in *A. ostoyae* and *A. cepistipes*
