# Supplementary material for: Hallmarks of Basidiomycete Soft- and White-Rot in Wood-Decay -Omics Data of Two Armillaria Species
Source: Microorganisms. 2021 Jan 11;9(1):149. doi: 10.3390/microorganisms9010149 (PMC7827401; doi:10.3390/microorganisms9010149)
Supplement: Supplementary file 1 [file microorganisms-09-00149-s001.zip › Supplementary file_Info.pdf]

Supplementary Material for

Hallmarks of basidiomycete soft- and white-rot in wood-decay -omics data of two *Armillaria* species

Neha Sahu[1,2], Zsolt Merényi[1], Balázs Bálint[1], Brigitta Kiss[1], György Sipos[3,4], Rebecca Owens[5], László G. Nagy[1,6]*

This file contains:

Supplementary Figure 1.

Supplementary Figure 2.

Supplementary Figure 3.

Supplementary Figure 4.

Supplementary Figure 5.

Other supplementary files:

Supplementary Table 1. RNA-Seq mapping statistics for *A. ostoyae* and *A. cepistipes*

Supplementary Table 2. Differentially expressed genes (RNA-Seq) in *A. ostoyae* and *A. cepistipes*

Supplementary Table 3. Differentially abundant proteins (Proteomics) in *A. ostoyae* and *A. cepistipes*

Supplementary Table 4. List of enriched GO terms in *A. ostoyae* and *A. cepistipes* in the transcriptomics and proteomics analyses

Supplementary Table 5. Single copy co-orthologs in both species, with common and species-specific DEGs/DAPs in *A. ostoyae* and *A. cepistipes*

Supplementary Table 6. Carbohydrate-active enzymes (CAZymes) and plant cell wall degrading enzymes (PCWDEs) identified in transcriptomics and proteomics analyses of *A. ostoyae* and *A. cepistipes*

Supplementary Table 7. Putative transporters identified in transcriptomics and proteomics analyses of *A. ostoyae* and *A. cepistipes*

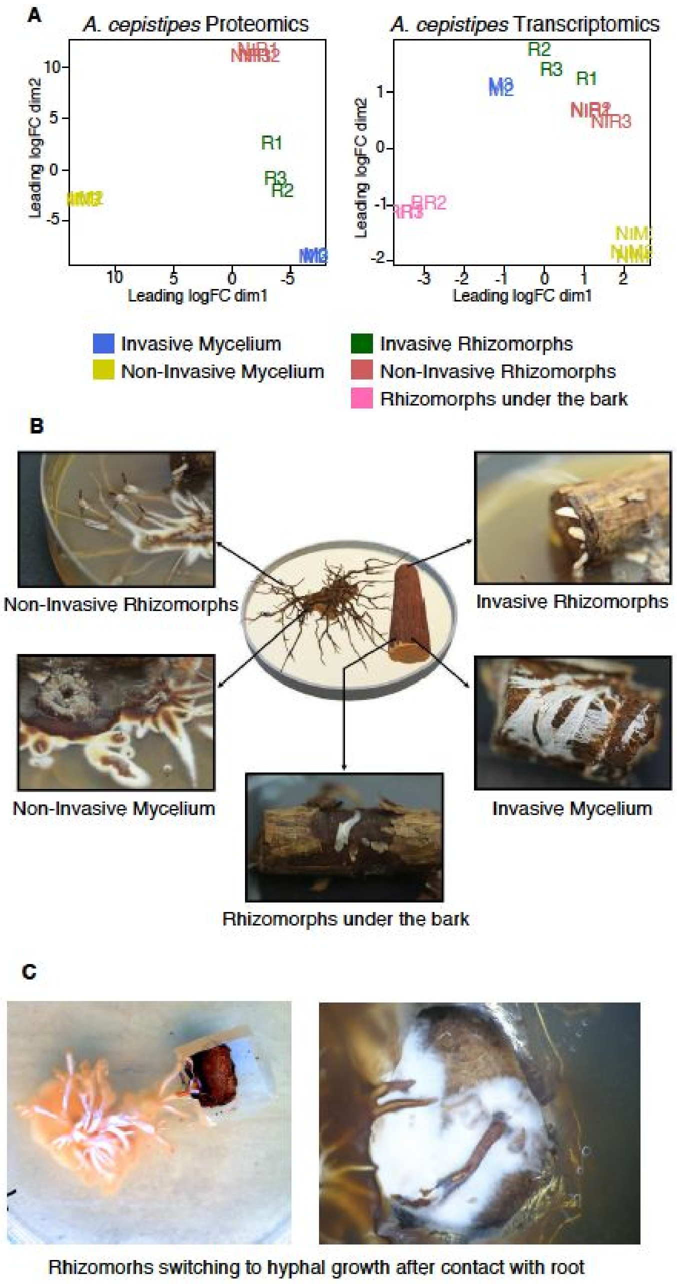

Supplementary Figure 1. Experimental setup. A) Multidimensional scaling of three biological replicates from each of the tissue types in *A. cepistipes* for proteomics (left) and transcriptomics (right). B) The four tissue types sampled for transcriptomics and proteomics analysis viz. invasive mycelium (growing beneath the outer layer of the root), invasive rhizomorphs (emerging out of the roots), non-invasive mycelium and non-invasive rhizomorphs (growing in absence of root), along with additional RR (rhizomorphs growing beneath the outer layer of the root) in *A. cepistipes*. C) Pictures showing rhizomorphs differentiating into hyphae in contact with the spruce root.

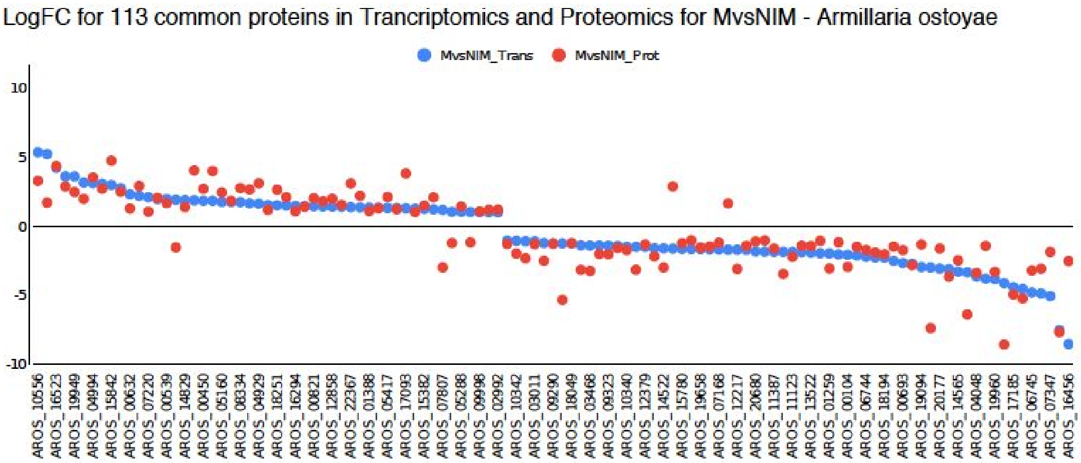

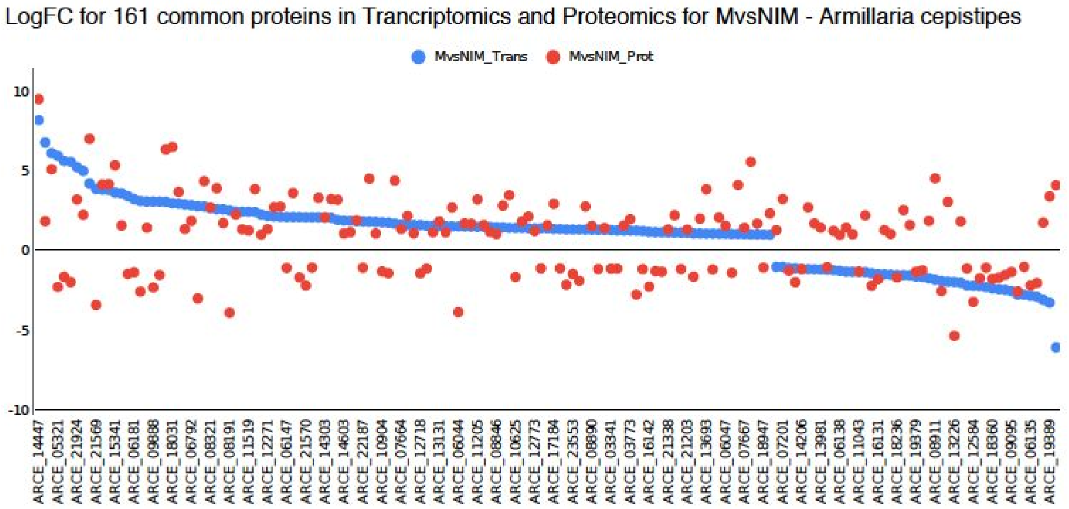

Supplementary Figure 2. Log fold changes in MvsNIM of genes identified in both transcriptomics and proteomics data in *A. ostoyae* (top) and *A. cepistipes* (bottom). The logFC for transcripts (blue) are arranged from increased to decreasing order, overlaid with logFC from proteomics (red) shows a limited correlation between the two omics approaches.

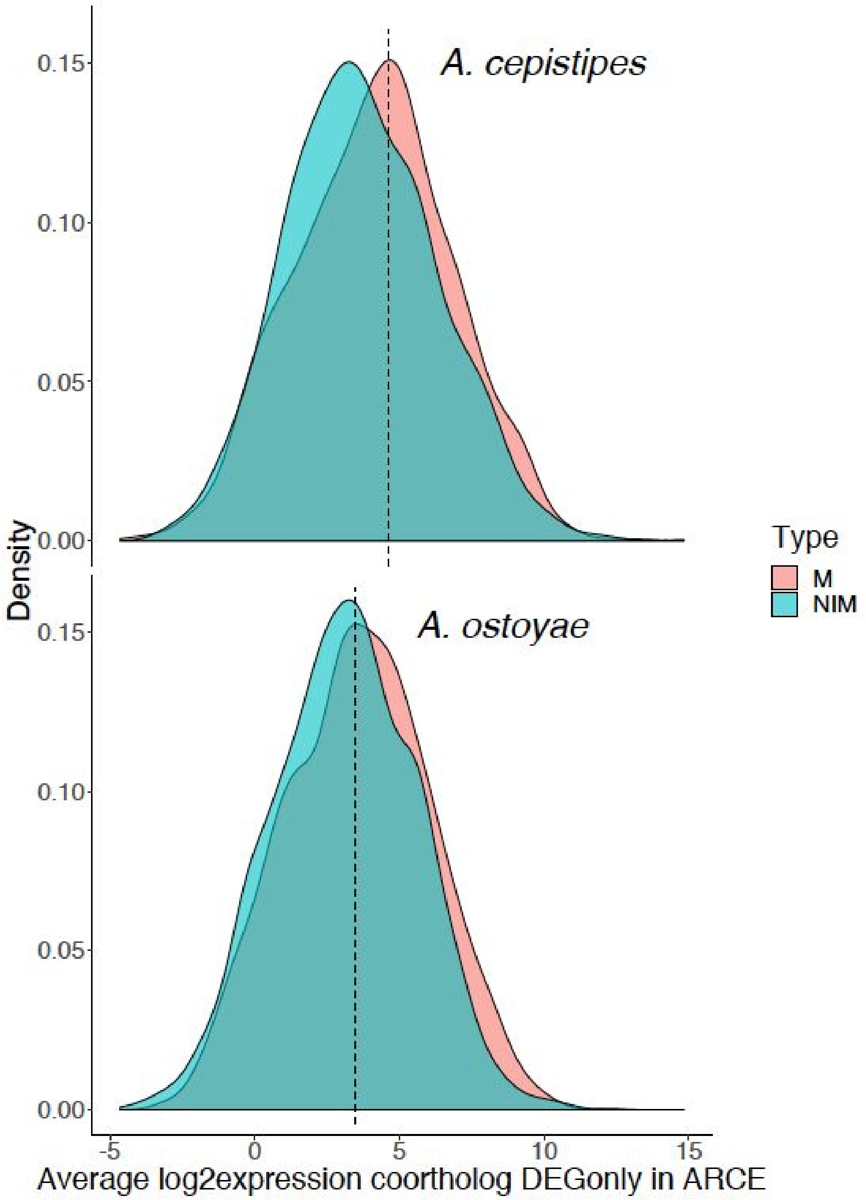

Supplementary Figure 3. Distribution of raw expression values in both species for co-orthologs differentially expressed in MvsNIM of *A. cepistipes*. Baseline expression of genes in non-invasive mycelia of *A. ostoyae* was not higher than that in A. cepistipes, indicating a stronger response of *A. cepistipes*.

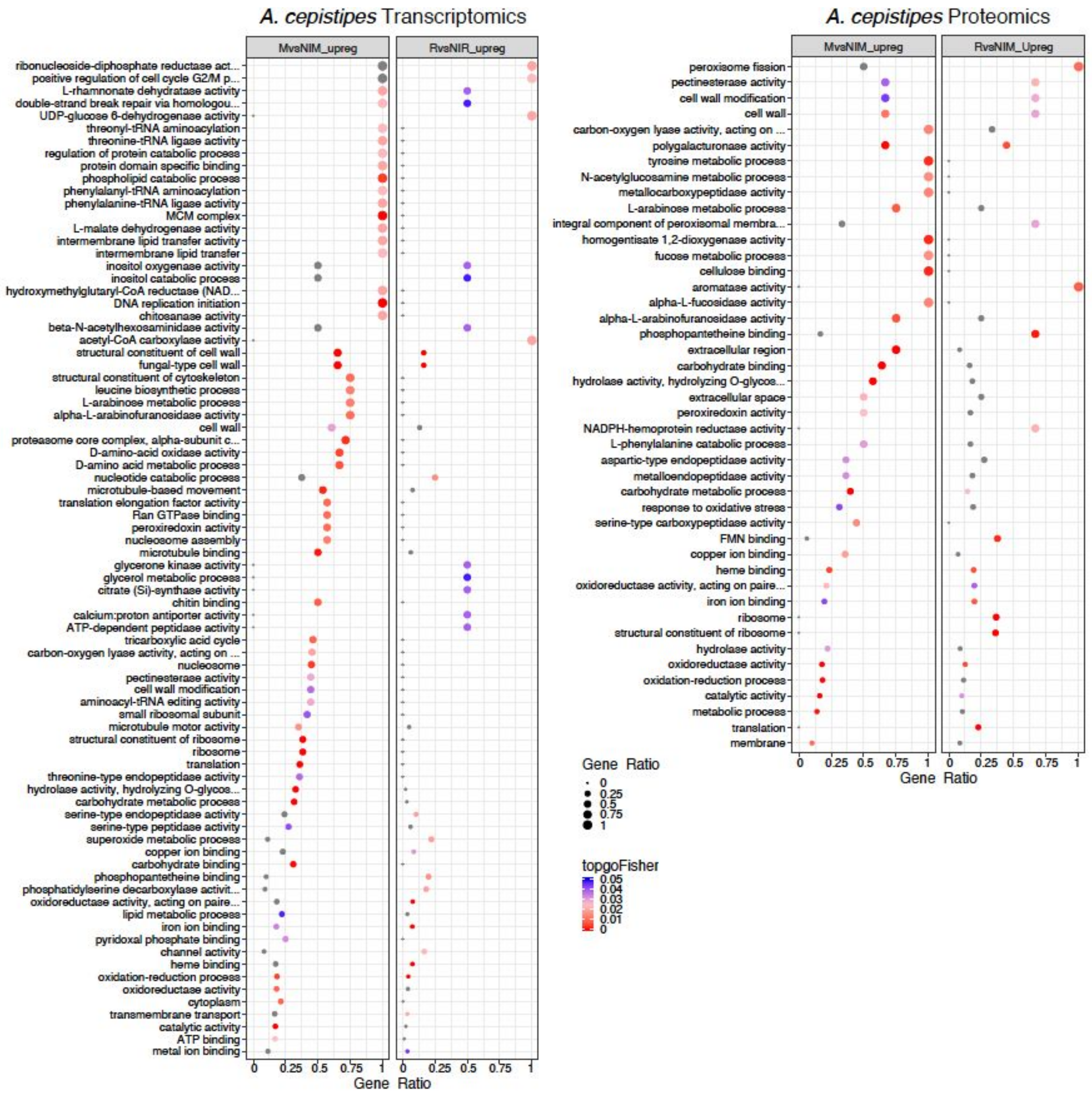

Supplementary Figure 4. Enriched GO terms in M*vs*NIM and R*vs*NIR of *A. cepistipes* for transcriptomics (left) and proteomics (right). The ratio of number of a particular GO term in a specific comparison (mycelium vs non-invasive mycelium or in rhizomorphs vs non-invasive rhizomorphs) to the total number of that GO term for a species was used to plot gene ratios for enriched GO terms (p<0.05, Fisher's exact test). The size of the dot is directly proportional to gene ratio, and the color of the dots corresponds to p-values. Grey dots represent GO terms, enriched in only one of the comparisons *i.e* either mycelium vs non-invasive mycelium or rhizomorphs vs non-invasive rhizomorphs.

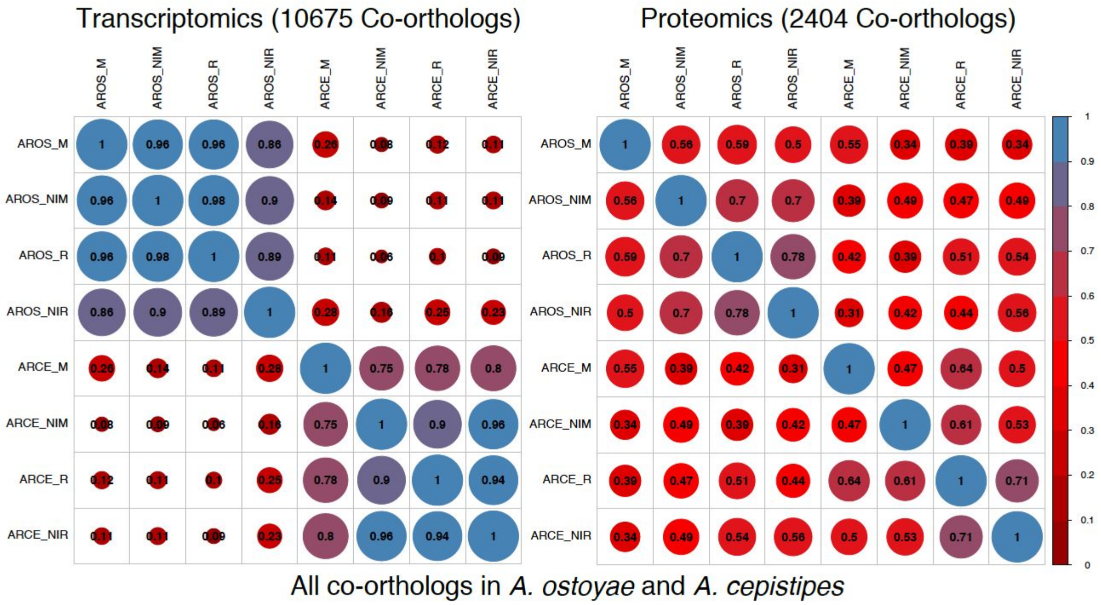

Supplementary Figure 5. Correlogram for all co-orthologs in the two species, showing the correlation between samples across the two species. Blue represents a higher correlation and red represents lower. The size of the circle is directly proportional to a higher correlation. Pairwise mean Pearson correlation coefficients are indicated as numbers in the circles.
