# Supplementary figures and images for: Hallmarks of Basidiomycete Soft- and White-Rot in Wood-Decay -Omics Data of Two Armillaria Species
Source: Microorganisms. 2021 Jan 11;9(1):149. doi: 10.3390/microorganisms9010149 (PMC7827401; doi:10.3390/microorganisms9010149)

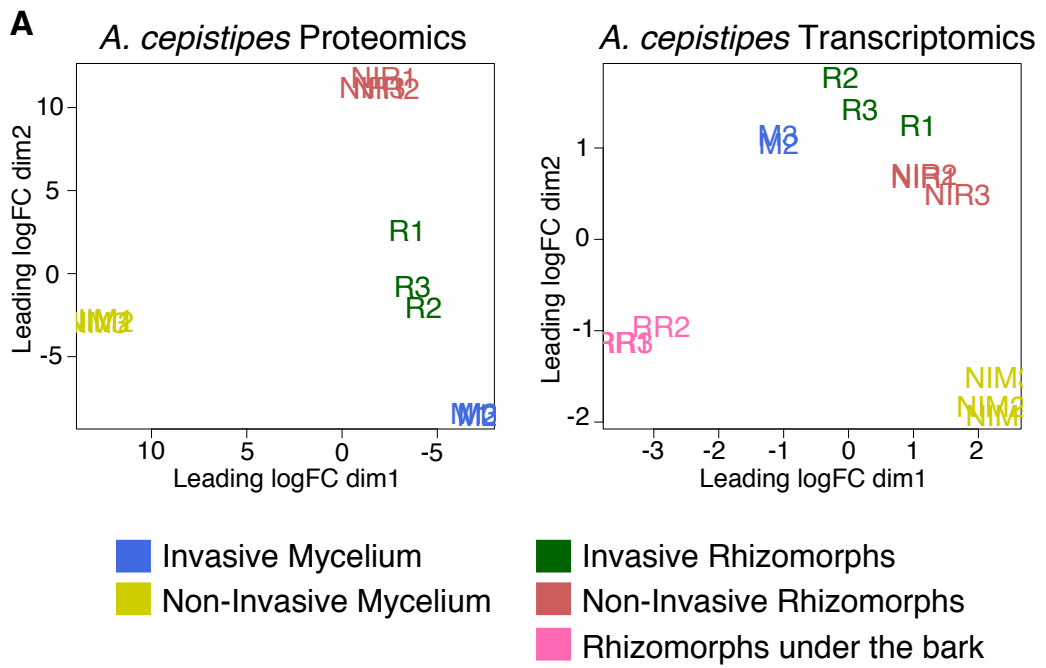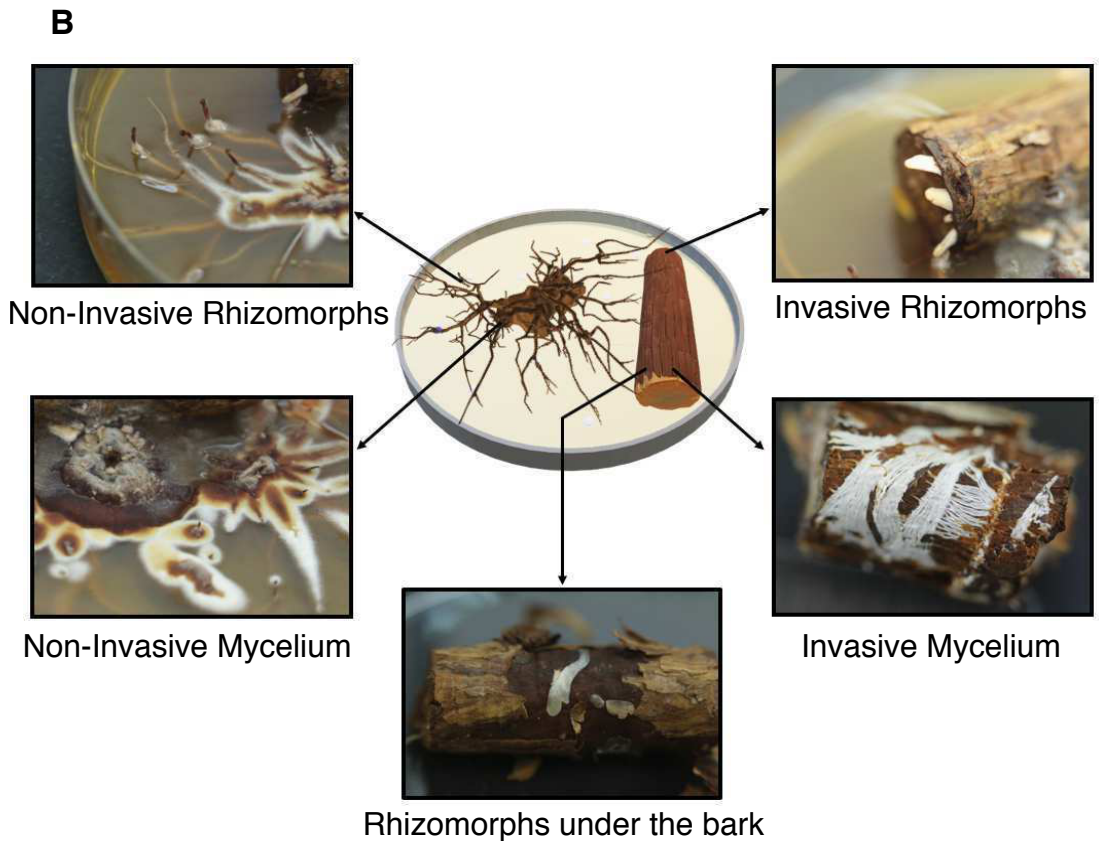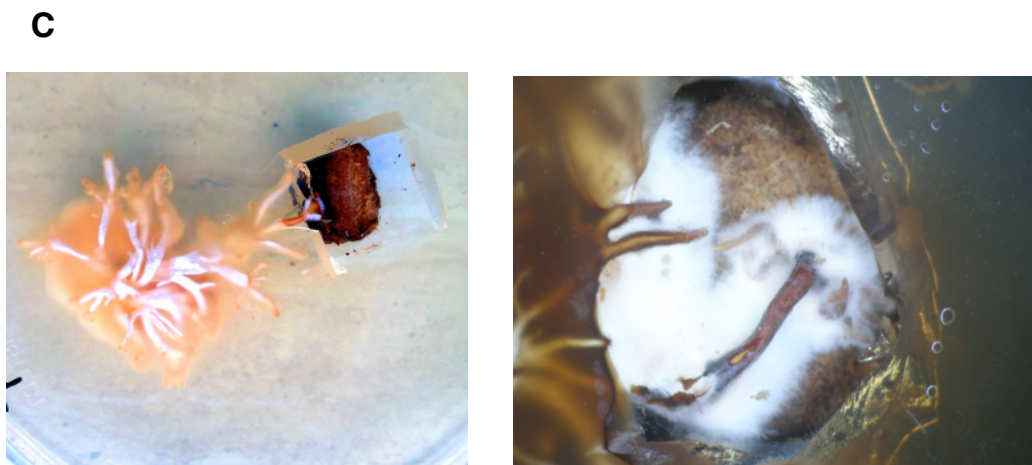

Rhizomorphs switching to hyphal growth after contact with root

Supplement: Supplementary file 1 [file microorganisms-09-00149-s001.zip › Supplementary Figure 1.pdf]

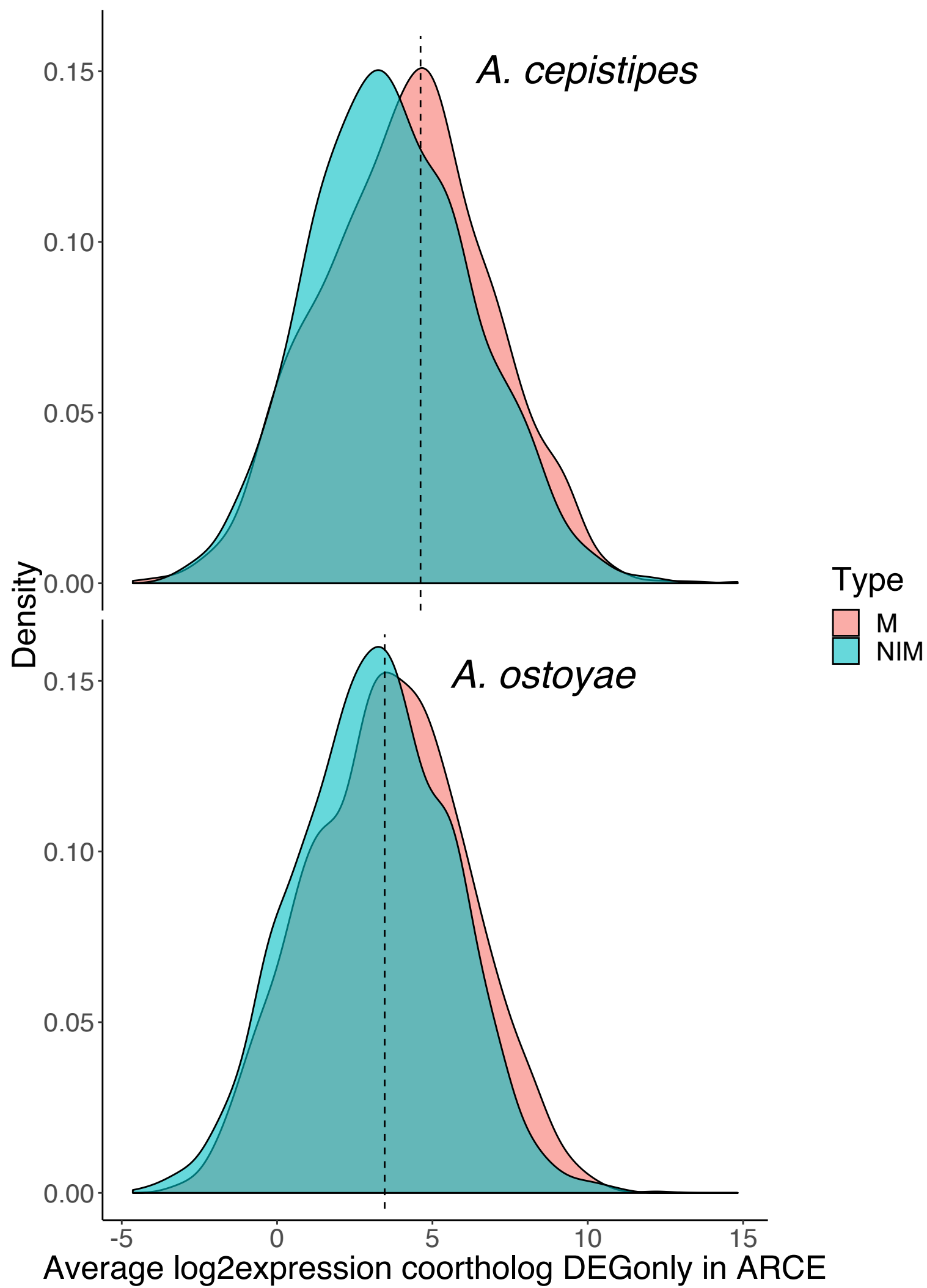

Supplement: Supplementary file 1 [file microorganisms-09-00149-s001.zip › Supplementary Figure 3.pdf]

*A. cepistipes* Transcriptomics

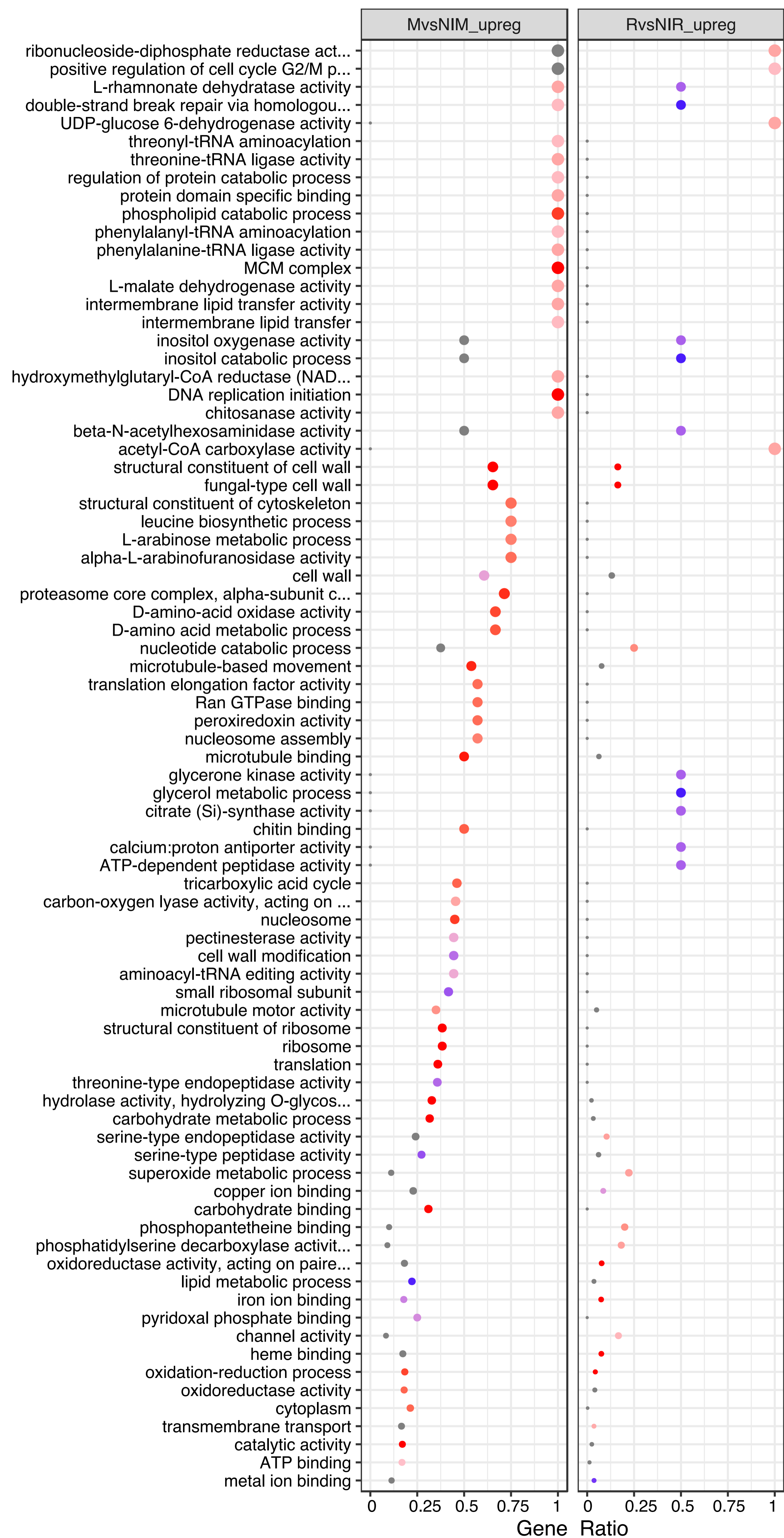

*A. cepistipes* Proteomics

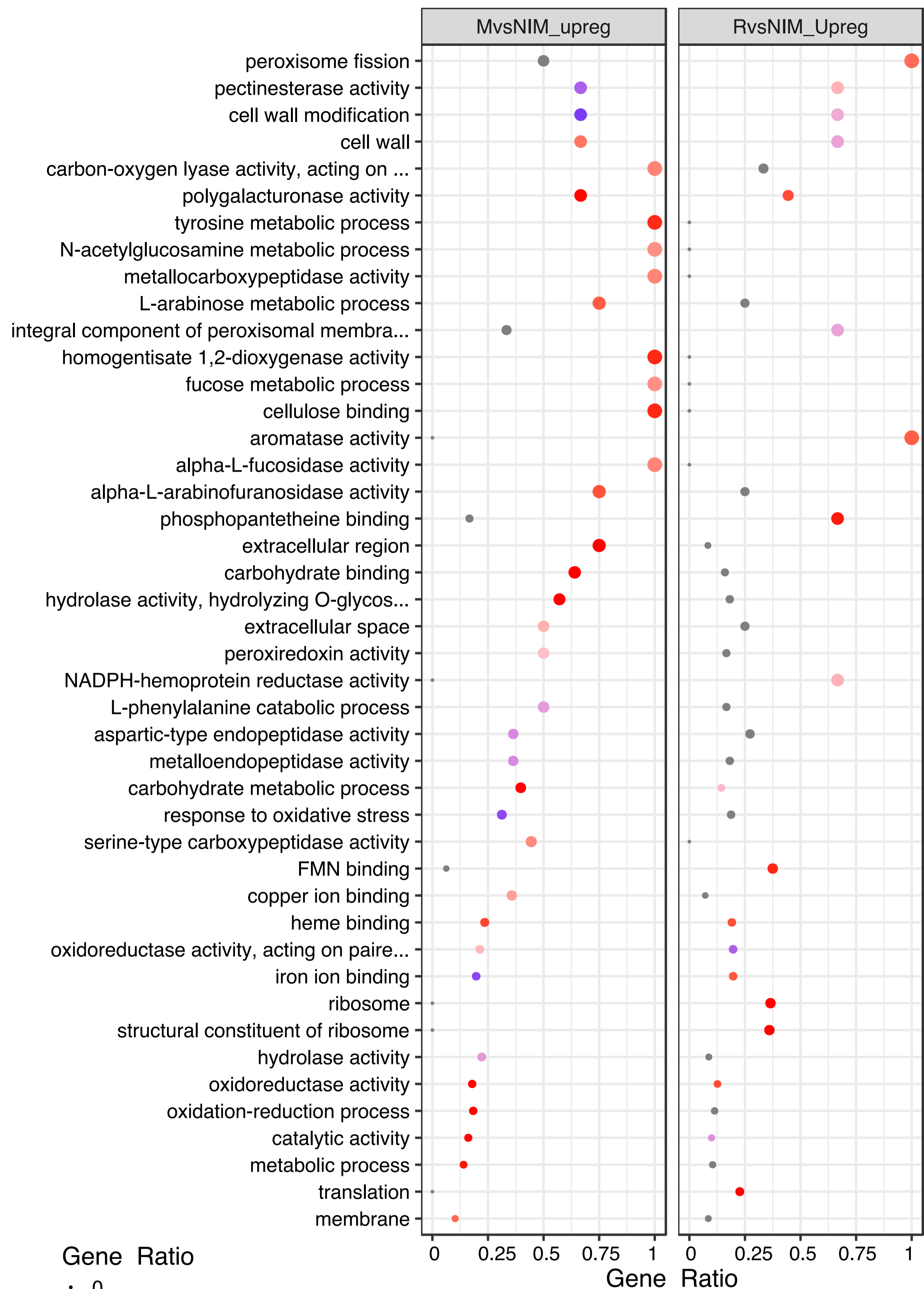

Supplement: Supplementary file 1 [file microorganisms-09-00149-s001.zip › Supplementary Figure 4.pdf]
